# Quantitative Analysis of Houseflies-mediated Food Contamination with Bacteria

**DOI:** 10.14252/foodsafetyfscj.2018013

**Published:** 2019-03-29

**Authors:** Akira Fukuda, Masaru Usui, Chinami Masui, Yutaka Tamura

**Affiliations:** Laboratory of Food Microbiology and Food Safety, School of Veterinary Medicine, Rakuno Gakuen University, 582 Midorimachi, Bunkyodai, Ebetsu, Hokkaido 069-8501, Japan

**Keywords:** food contamination, food safety, housefly, vector

## Abstract

Flies play a key role as vectors in transmitting various bacteria and pose bacterial contamination risk to food. To evaluate the time- and concentration-related bacterial contamination of food by houseflies based on their attraction to the food, we determined the number of fed antimicrobial-resistant *Escherichia coli* transferred from houseflies to foods, sugar and milk mixture, apple, and castella (such as sponge cake). Houseflies contaminated the foods with the fed *E. coli* within 5 min, and the bacteria were present in high numbers on apple and castella (3.3 × 10^3^ and 3.5 × 10^4^ CFU/g of food, respectively). Furthermore, the number of fed *E. coli* on the foods increased with time, rising to 3.6 × 10^4^–1.7 × 10^5^ CFU/g. We show that the food contamination level caused by houseflies depends on the concentration of bacteria that the houseflies carry, the contact time with the food, and the attraction of the flies to the food.

## Introduction

Filth flies, such as the non-binding flies [Muscidae (housefly) and Calliphoridae (blowfly)], are frequently found in a wide range of habitats. As vectors, they carry and transmit a great variety of bacteria including antimicrobial-resistant and pathogenic bacteria, from numerous sources owing to their strong flight ability^1^^,^^2^^)^. The presence of flies indicates the palatability of various foods. Flies pose food contamination risk because of the bacteria they carry from the surfaces by touch, in their vomit from the crop, and in the excretion products from their alimentary canal^3^^)^. This potential contamination risk indicates the possibility of flies transmitting bacteria to humans through food^4^^)^. However, the extent of food contamination caused by flies is uncertain, because it depends on the bacterial concentration they carry and their contact time with the food, especially over short periods. Therefore, to determine the degree of bacterial contamination of several types of food caused by flies and their perceived palatability of those foods, we quantified the levels of bacterial contamination of foods over time using houseflies that had fed varying concentrations of *Escherichia coli*.

## Materials and Methods

### Houseflies

The Chemical Specialties Manufacturer’s Association (CSMA) housefly strain (*Musca domestica*) was provided by Sumika Life Tech. Ltd. (Osaka, Japan), and maintained for multiple generations in nets at 25°C under a 14:10 h light:dark cycle in an isolator. The houseflies were provided with distilled water and a mixture of the same amount of skim milk and sugar (MS)^5^^)^.

### Bacterial Feeding of Houseflies

Approximately 130 adult houseflies (5–7 days after emergence) were transferred to a cage (15 × 15 × 90 cm) and were fed a suspension culture of cefotaxime-resistant *E. coli* (Strain No. 133) for 3 h at 25°C *ad libitum*^5^^,^^6^^)^. The fed *E. coli* harbored IncFIB plasmid containing the *bla*_CTX-M-15_, *bla*_TEM_ and *tetA* gene, and belong to phylogenic group D. Multilocus sequence typing of this strain was ST38 which was commonly found in human and livestock, especially in extended-spectrum β-lactamase-producing *E. coli* in Japan^6^^,^^7^^)^. To prepare the bacterial suspension, the *E. coli* was grown in tryptic soy broth (Bacto Peptone, BD Biosciences, Franklin Lakes, NJ, USA) at 37°C for 14–18 h, and then centrifuged for 15 min at 1,800 ×*g*. The supernatant was discarded, and the cell pellet was suspended in a sterilized milk and sugar solution (115°C for 20 min) to adjust the bacterial number to the desired level^5^^,^^8^^)^. The houseflies were provided *E. coli* suspension with the high, intermediate, and low number of bacteria (10^9^, 10^7^, and 10^4^ colony-forming unit (CFU)/mL, respectively) in 35 mm sterile dishes for feeding. As such, housefly specimens containing high (HI-fly), intermediate (IM-fly), and low (LO-fly) numbers of fed bacteria were prepared.

### Houseflies-medicated Food Contamination

After bacterial feeding to the houseflies, the food contamination experiment was performed by transferring 30 houseflies into each of the four new cages, which were provided with 2 g each of MS, apple, and castella (a sponge cake containing flour, sugar, and egg) in sterile dishes. We collected the houseflies and the food items after leaving them for 0, 5, 10, 30, and 60 min together in the cages. They were immediately processed for quantification of bacterial contamination on food items from houseflies.

### Quantification of Bacteria

Five houseflies were pooled from each group, and the 2 g of food were homogenized in 2.5 mL of phosphate-buffered saline (PBS) using a mortar and pestle. Then the fed strain was quantified by performing serial dilution and plating samples on deoxycholate hydrogen sulfide lactose (DHL) agar (Nissui Pharmaceutical, Tokyo, Japan) supplemented with 4 μg/mL cefotaxime (Sigma-Aldrich, St. Louis, MO, USA)^5^^)^. After incubation for 24 h at 37°C, the colonies were counted to obtain CFU of bacteria. The experiment was repeated five times and the data are expressed as the mean ± standard deviation. On these plates, the colonies correspond to the fed bacteria were not detected in the houseflies and the foods.

Statistical significance was determined using a Mann-Whitney *U* test, with the significance threshold set at *p* < 0.05.

## Results and Discussion

### Viability of Fed Bacteria in Houseflies

After feeding high, intermediate, and low concentrations of the bacterial suspensions, the houseflies harbored the fed *E. coli* strain for 60 min at concentration ranges of 1.6 × 10^6^–1.4 × 10^8^ CFU/HI-fly, 1.9 × 10^4^–1.7 × 10^5^ CFU/IM-fly, and 5.5 × 10^1^–8.0 × 10^2^ CFU/LO-fly, respectively. The concentrations of the fed strain in the houseflies reflected those in the original bacterial suspensions. In field studies, various species of bacteria including *E. coli* have been isolated from flies in concentration ranges of 10^1^–10^6^ CFU/fly^3^^)^, which is similar to the levels observed in our experiment. Furthermore, these bacteria were maintained in flies for a specific period that depended on the fed bacterial concentrations^3^^)^.

### Houseflies-medicated Bacterial Contamination of Foods

**Fig. 1.**
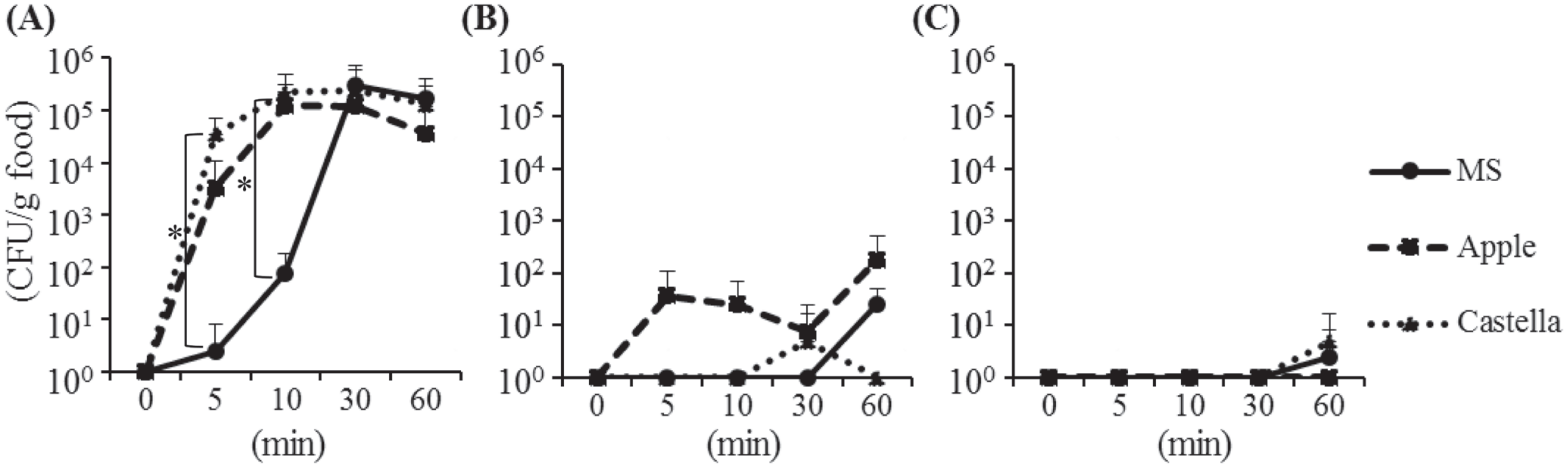
Food contamination by houseflies that fed *Escherichia coli* strain. Houseflies were provided with (A) high (10^9^ CFU/mL), (B) intermediate (10^7^ CFU/mL), and (C) low (10^6^ CFU/mL) concentrations of the bacterial suspension. * *p* < 0.05. MS, a mixture of sugar and milk

In the food contamination experiment, the food items were contaminated with the fed *E. coli* strain in the houseflies which carried fed bacteria at similar concentration levels to those observed in the field (Fig. 1). The concentrations of the fed strain in the foods also correlated with those in the houseflies. The HI-fly group contaminated all the food types within 5 min, especially the castella showed high bacterial numbers compared with MS at 5 and 10 min (*p* < 0.05). Furthermore, the concentrations of the fed strain on the foods increased with time (Fig. 1A). Eventually, at 60 min, the fed strain contaminated the food at a range of 3.6 × 10^4^–1.7 × 10^5^ CFU/g. The IM-fly group contaminated the apple within 5 min, and the fed bacteria by the IM- and LO-fly groups were detected in the castella and MS within 30 or 60 min (*p* > 0.05) (Fig. 1B and C). These results suggest that flies carrying high concentrations of bacteria show increased levels of bacterial contamination of foods.

Houseflies carrying high number of bacteria contaminate food in a short time. Previous studies showed that bacterial contamination of food by flies was caused by direct contact, and approximately 10^3^ CFU were transmitted from flies per landing^9^^,^^10^^)^. The number of flies and landings on foods correlated with those of bacterial levels in the foods^4^^,^^10^^)^. To reduce bacterial contamination of food by flies, preventing their direct contact using protective barriers such as nets around food is an effective strategy^9^^,^^11^^)^.

For the LO-fly group, the fed bacteria on the foods were under the limit of detection (25 CFU/g) for up to 60 min. However, food-borne pathogenic bacteria, such as *E. coli* O157, can infect humans with an extremely small number of organisms, and can also proliferate in foods^12^^,^^13^^)^. Flies have been shown to transmit such pathogenic bacteria^1^^,^^3^^)^, and even low numbers of bacteria carried by flies are a potential risk of causing food-borne diseases.

In our experiment, apple and castella were more rapidly contaminated than the MS. Polyphagy is a feeding habit of flies^9^^,^^13^^)^. They are attracted to foods for various factors, including the presence of volatile compounds^14^^,^^15^^,^^16^^)^. Our results suggest that the flies are attracted to some foods more than the others was influenced and, therefore, highly attractive foods have increased risk of bacterial contamination by flies.

In conclusion, flies contaminate foods at a rate and level that depends on the concentration of bacteria they carry, contact time with the food, and the attraction of flies to the food for reducing risk of bacterial contamination from both surface and intestine of flies. Therefore, it is important to prevent physical contact of flies with foods even for a brief time. Furthermore, it is important to maintain a clean environment to reduce adverse bacteria, such as antimicrobial-resistant and pathogenic bacteria, which could be transferred to humans by the flies that carry them. Finally, to ensure food safety and prevent bacterial contamination in food settings, hygiene management including the control of insects is important.
